# A Modified Extended Kalman Filter for a Two-Antenna GPS/INS Vehicular Navigation System

**DOI:** 10.3390/s18113809

**Published:** 2018-11-06

**Authors:** Yushi Hao, Aigong Xu, Xin Sui, Yulei Wang

**Affiliations:** 1 School of Geomatics, Liaoning Technical University, Fuxin 123000, China; kinghaoys@163.com (Y.H.); survey_suixin@163.com (X.S.); 2 State Key Laboratory of Satellite Navigation System and Equipment Technology, Shijiazhuang 050081, China; 3 State Key Laboratory of Automotive Simulation and Control, Jilin University, Changchun 130025, China; wangyulei@jlu.edu.cn

**Keywords:** two-antenna GPS/INS, navigation system, adaptive noise covariance, measurement outliers, positive feedback, numerical issue

## Abstract

Recently, the integration of an inertial navigation system (INS) and the Global Positioning System (GPS) with a two-antenna GPS receiver has been suggested to improve the stability and accuracy in harsh environments. As is well known, the statistics of state process noise and measurement noise are critical factors to avoid numerical problems and obtain stable and accurate estimates. In this paper, a modified extended Kalman filter (EKF) is proposed by properly adapting the statistics of state process and observation noises through the innovation-based adaptive estimation (IAE) method. The impact of innovation perturbation produced by measurement outliers is found to account for positive feedback and numerical issues. Measurement noise covariance is updated based on a remodification algorithm according to measurement reliability specifications. An experimental field test was performed to demonstrate the robustness of the proposed state estimation method against dynamic model errors and measurement outliers.

## 1. Introduction

High-accuracy positioning is an important issue for vehicular navigation applications. With the rapid development of the multiple-constellation Global Navigation Satellite System (multi-GNSS) [1], single-frequency real-time kinematics has better performance on centimeter-level positioning due to the increasing satellite visibility and better spatial geometry [2,3,4]. However, real-time kinematic (RTK) performance degrades in harsh environments with frequent signal blockages, multipaths, and even multiple constellations. Therefore, an increasing number of studies have focused on the integrated navigation system of the Global Positioning System (GPS) and Inertial Navigation System (INS) due to their complimentary characteristics, enabling such integrated systems to also provide accurate and continuous navigation information (e.g., position, velocity, and attitude) in harsh measurement environments [5,6,7,8].

Usually, the extended Kalman filter (EKF) is well-established to accomplish data fusion of an integrated system [9], and the GNSS/INS integration algorithm normally uses a Kalman filter to fuse data [10]. However, its performance strongly relies on the accuracy of the statistics of state parameters and measurements, for example, described by their noise probability density function (PDF). In practice, it is almost impossible to obtain such accurate information in advance. Therefore, the state estimates from a conventional EKF suffer from uncertain perturbations when the covariance matrix of measurement noise R and/or the covariance matrix of process noise Q do not match the actual situation [11]. Particle filtering (PF) overcomes issues concerning inaccurate PDF and becomes an effective method for processing nonlinear non-Gaussian systems [12,13]. PF is a sequential Monte Carlo estimation algorithm with a great advantage in solving non-Gaussian issues since it can randomly generate a large number of random samples for describing PDF, adjusting particle weight and sample position to approximate the posterior PDF. Therefore, the major advantage of particle filtering is that it can model any form of probability distribution [14,15]. However, its drawbacks, such as a large amount of calculation, difficulty of PDF selection, and particle degradation in harsh measurement environments are also hard to accept, and an absolutely effective adjusting method to address them has not been found. In vehicular navigation application, relatively accurate PDFs of state estimate predictions and measurements are hardly generated by the filter itself due the frequently occurring measurement outliers without boundedness and periodicity. Hence, one of the main factors affecting filter performance is the controller and measurement outlier or fault detection, isolation, and reconfiguration [16,17,18]. To this end, a considerable portion of scholars have developed optimal adaptive Kalman filters (AKFs) to determine the most appropriate weight between Q and R based on the maximum likelihood criterion where the Kalman gain factors for accuracy improvement are based on the treatment of variable error characteristics [19,20,21]. In an AKF, the adaptive process noise covariance and measurement noise covariance can be tuned by a scalar adaptive factor based on the analysis of the predicted residual vector. The multiple-model-based adaptive estimation (MMAE) method and innovation-based adaptive estimation (IAE) method are the most representative methods in this category [22]. The IAE method has a lower computational burden than MMAE due to the use of a single filter [22].

Furthermore, both robust and adaptive robust Kalman filters [23,24,25,26,27,28] have been discussed regarding controller and measurement outliers. These approaches can lead to better performance [29,30,31,32,33,34] in term of robustness and adaptivity due to the IAE method and corresponding equivalent weight matrix derived from the Huber function [35]. However, they could be further improved, especially under the situations that the statistics of both measurement and state noise have to be adapted.

In the framework of GPS/INS integration, in order to provide accurate attitude information especially under movement with low acceleration, a multiantenna receiver is suggested [36,37] since orientation can be derived from both the GPS baseline vector, and that provided by the INS [38,39]. The performance of this approach relies on the relative accuracy of the baseline vector, i.e., a yaw error of 2.3° occurs for a baseline of 0.5 m with a position accuracy of 2 cm. However, a GPS-estimated baseline vector of low precision of several centimeters may result in unstable or biased state estimates if measurement noise is not properly adapted. In some cases, state estimates can even experience positive feedback from the measurement noise through the system model, rapidly diverging from the true counterparts [40]. State uncertainties are often added (typically twice or three times) to reduce the impact of measurement outliers. However, in this approach, the magnitude of attitude error is relatively small, filter-updating performance can be degraded, and the benefit of the augmented attitude error equation cannot be exerted.

Furthermore, there are mainly two different integration modes: loosely coupled (LC) and tightly coupled (TC) integration. In LC integration, which is the most common type of integration, measurement vectors are constructed based on the difference between solutions calculated by the GPS and INS, whereas TC integration directly utilizes the GPS pseudorange or carrier-phase observations. TC integration is more advantageous because INS measurements could improve GPS processing in terms of ambiguity resolution (AR) [40,41]. However, state estimates using TC integration may face serious problems in terms of the reliability of raw GPS measurements according to References [9,42], because the prior acknowledgement measurement quality of the single-epoch phase carrier and pseudorange (satellite elevation, signal-to-noise ratio) is relatively limited, which means that a KF implemented with TC integration is more sensitive to the quality of raw GPS measurements compared with a KF implemented with LC integration. As a comparison, when adopting LC integration, GPS performance in terms of positioning and measurements can be noticed, which is considered the basis for fault detection in filter measurement updating.

To address the issues concerning the dynamic model errors and, in particular, measurement outliers, we propose an adaptive robust approach for two-antenna GPS/INS LC integration in which the IAE method is utilized to design a factor for the adaptive covariance. Several equations concerned with IAE drawbacks are derived to account for and overcome the numerical and feedback issues of two-antenna GPS/INS integration caused by measurement outliers and unknown state uncertainties, and an adaptive reconfiguration for measurement noise covariance according to measurement reliability is designed. The desirable properties of the proposed approach are summarized as follows:

- Adaptive modification of noise covariance can treat dynamic model errors and measurement disturbance to reduce their impact on state estimation, especially when the statistics of both measured and predicted noise have to be adapted. With filter updating, positive feedback and numerical issues can be reduced by quantifying statistical measurement noise on a more granular level based on the corresponding quantifications of measurement reliability in the case of measurement outliers.
- The proposed method can accurately quantify measurement reliability. It is an evidence-based regulation method with the benefit of attenuating the impact of innovation perturbation. In addition to the assured stability of filter updating, the performance of the augmented measurement equation in state error feedback for precious measurements is improved.

The paper is organized as follows. An overview of the two-antenna GPS/INS-integrated algorithm is provided in Section 2. In Section 3, the adaptive modification of noise covariance is discussed. The field experiment and results for different schemes are compared in Section 4 to verify the superiority of the proposed approach compared to existing methods. Finally, several conclusions of this work are drawn.

## 2. Two-Antenna GPS/INS

### 2.1. Inertial Dynamic Model

The inertial dynamic model is derived from the Psi-Angle error model based on INS error differential equations and summarized as [43]:

(1) $$\begin{array}{l} \delta \dot{r}=-\omega_{\mathrm{en}}\times \delta r+\delta v\\ \delta \dot{v}=f\times \psi -(2\omega_{\mathrm{ie}}+\omega_{\mathrm{en}})\times \delta v+\delta g+C_{b}^{n}\delta f^{b}\\ \;\dot{\psi }=-(\omega_{\mathrm{ie}}+\omega_{\mathrm{en}})\times \psi -C_{b}^{n}\delta \omega^{b} \end{array}$$

where δ denotes the corresponding error or uncertainty of the vectors, $\dot{•}$ denotes the first derivatives, × denotes the cross-product of two vectors, δr, δv, and ψ are the position, velocity, and attitude error state vectors, respectively, f is the specific force vector, $\omega_{\mathrm{ie}}$ is the earth rotation vector, and $\omega_{\mathrm{en}}$ is the craft-rate vector [44]. $\delta f^{b}$ and $\delta \omega^{b}$ are the accelerometer error and gyroscope error, respectively, and are written as:

(2) $$\begin{array}{c} \delta f^{b}=b_{a}+\mathrm{diag}(f^{b})s_{a}\\ \delta \omega^{b}=b_{g}+\mathrm{diag}(\omega^{b})s_{g} \end{array}$$

where diag(•) denotes the diagonal form of the matrix, and $b_{a}$ and $s_{a}$ are the accelerometer bias and accelerometer scale factor vectors, respectively. $b_{g}$ and $s_{g}$ are the gyroscope bias and gyroscope scale factor vectors, respectively. The inertial measurement unit (IMU) sensor error terms ε, such as bias and scale factors, are modeled as first-order Gauss–Markov (GM) processes:

(3) $$\dot{\varepsilon }=-T^{-1}\varepsilon +w_{\varepsilon }$$

where T is the correlation time, $w_{\varepsilon }$ is the corresponding process noise vector, and δg is the gravity uncertainty error vector, projected as:

(4) $$\delta g=\mathrm{diag}(-\omega_{s}^{2}-\omega_{s}^{2}\;2\omega_{s}^{2})\delta r$$

where $\omega_{s}$ denotes the Schuler frequency [43,44,45,46,47]. Subscript b indicates the body frame (b-frame) with vehicle axes, i.e., forward–transversal–down. Subscript n indicates the navigation frame (n-frame) is a local geodetic frame with the *x*-axis towards geodetic north, the *z*-axis towards an orthogonal to the reference ellipsoid pointing down, and the *y*-axis completing a right-handed orthogonal frame, i.e., north–east–down (NED). $C_{b}^{n}$ is the direction cosine matrix (DCM) from the body frame to the n-frame.

In this research, an INS error with 21 states was developed according to the equations mentioned above. Nine navigation parameters expressed in the n-frame, and 12 inertial sensor error parameters expressed in the b-frame, are involved. The complete error state sequence is expressed as:

(5) $$\delta x={\left(\delta r\;\delta v\;\psi \;b_{g}\;b_{a}\;s_{g}\;s_{a}\right)}^{T}$$

### 2.2. Measurement Model

**Figure 1:**
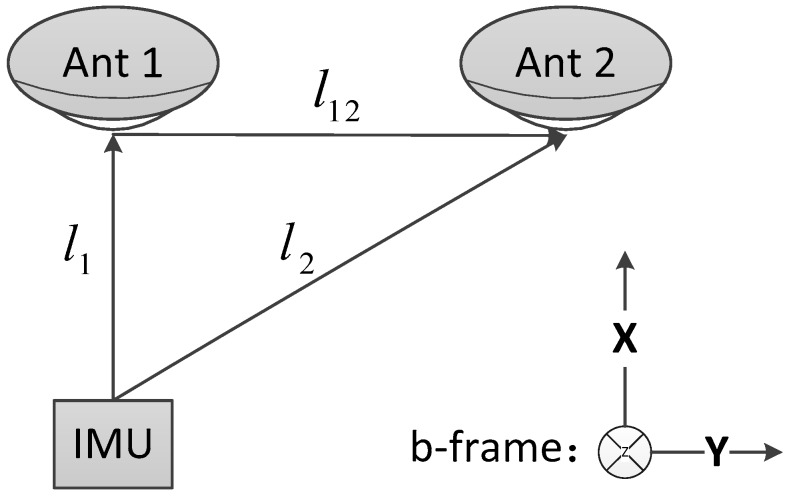
Physical-location relationship between the inertial measurement unit (IMU) and two Global Navigation Satellite System (GNSS) receiver antennas in the body frame.

Figure 1 illustrates the physical location relationship between the IMU and GNSS rover with two antennas (Ant 1 and Ant 2) in the b-frame.

Where $l_{i}$ is the level arm vector between the IMU and *i*-th antenna, while $l_{12}$ is the level arm vector between the two antennas, and can be expressed as:

(6) $$l_{12}=l_{2}-l_{1}$$

The linearized position error measurement equation is [43]:

(7) $$\Delta Z_{r}=r_{\mathrm{IMU}}-r_{\mathrm{GPS}1}+C_{b}^{n}l_{1}=\delta r+(C_{b}^{n}l_{1}\times )\psi -e_{{\tilde{r}}_{1}}$$

where Δ denotes the difference between measurements and predictions. The linearized velocity error measurement equation is [43]:

(8) $$\begin{array}{l} \Delta Z_{v}=(v_{\mathrm{IMU}}+(C_{b}^{n}(\omega^{b}\times )-(\omega_{\mathrm{in}}\times ))C_{b}^{n}l_{1}-v_{\mathrm{GPS}1})\\ \;=\delta v-((\omega_{\mathrm{in}}\times )C_{b}^{n}(l_{1}\times )+C_{b}^{n}((l_{1}\times \omega^{b})\times ))\psi +C_{b}^{n}(l_{1}\times )b_{g}+C_{b}^{n}(l_{1}\times )\mathrm{diag}(\omega^{b})s_{g}-e_{{\tilde{v}}_{1}} \end{array}$$

where ${•}_{\mathrm{IMU}}$ and ${•}_{\mathrm{GPS}}$ indicate that the corresponding vectors are obtained by the INS mechanization algorithm and GPS-RTK algorithm, respectively. (•×) denotes the skew symmetric matrix form of a vector. $e_{{\tilde{r}}_{1}}$ and $e_{{\tilde{v}}_{1}}$ are the corresponding measurement white-noise vectors. $\omega_{\mathrm{in}}$ is the rotation rate vector, and is expressed as:

(9) $$\omega_{\mathrm{in}}=\omega_{\mathrm{ie}}+\omega_{\mathrm{en}}$$

According to Equation (7), another position error measurement equation related to Rover 2 can be constructed as follows:

(10) $$\Delta Z_{r}=r_{\mathrm{IMU}}-r_{\mathrm{GPS}2}+C_{b}^{n}l_{2}=\delta r+(C_{b}^{n}l_{2}\times )\psi -e_{{\tilde{r}}_{2}}$$

where $e_{{\tilde{r}}_{2}}$ is the corresponding measurement white noise vector.

$l_{12}$ has already been accurately calibrated in the b-frame and is considered as a constraint that can be used for fast AR fixing, even for a low-cost single-frequency receiver. Assume that the two-antenna baseline vector $p_{12}^{e}$ in the earth frame (e-frame) can be accurately estimated by moving-reference-receiver GPS-RTK processing that a method used to determine relative position vector between the antennas mounted to a single platform. Then, the attitude error measurement equation can be constructed using the difference between Equations (7) and (10):

(11) $$\begin{array}{l} \Delta Z_{\psi }=r_{\mathrm{IMU}}+C_{b}^{n}l_{1}-r_{\mathrm{GPS}1}-(r_{\mathrm{IMU}}+C_{b}^{n}l_{2}-r_{\mathrm{GPS}2})\\ \;=C_{b}^{n}l_{12}-C_{e}^{n}p_{12}^{e}\\ \;=(C_{b}^{n}l_{12}\times )\psi -e_{{\tilde{r}}_{12}} \end{array}$$

where $e_{{\tilde{r}}_{12}}$ is the differential measurement white-noise sequence, and $C_{e}^{n}$ is the DCM from the e-frame to the n-frame.

Consequently, the ninth-order measurement error model of the proposed two-antenna GPS/INS integration is given by:

(12) ΔZ=Hδx+e

where ΔZ is the measurement vector, H is the measurement matrix calculated based on Equations (7), (8), and (11), e is the measurement white-noise sequence and, determined by GPS/RTKs, results in error variance that reflects measurement uncertainty.

(13) $$\begin{array}{l} \Delta Z={\left[\Delta Z_{r}\;\Delta Z_{v}\;\Delta Z_{\psi }\right]}^{T}\\ \;e={\left[e_{{\tilde{r}}_{1}}\;e_{{\tilde{v}}_{1}}\;e_{{\tilde{r}}_{12}}\right]}^{T}\\ \;H=\left[\begin{array}{ccccccc} I_{3} & 0 & C_{b}^{n}(l_{1}\times ) & 0 & 0 & 0 & 0\\ 0 & I_{3} & \left(\omega_{\mathrm{in}}\times )C_{b}^{n}(l_{1}\times )+C_{b}^{n}((l_{1}\times \omega^{b})\times \right) & C_{b}^{n}(l_{1}\times ) & 0 & C_{b}^{n}(l_{1}\times )\mathrm{diag}(\omega^{b}) & 0\\ 0 & 0 & C_{b}^{n}(l_{12}\times ) & 0 & 0 & 0 & 0 \end{array}\right] \end{array}$$

where $I_{3}$ is the third-order unit matrix, 0 is third-order zero matrix.

## 3. Adaptive Noise Covariance

As noted in the introduction, KFs have been widely used in data fusion, and their performance relies on the accuracy of the dynamic and measurement models, and the statistical accuracy of the noise covariance (Q and R). Fortunately, Q and R can still be adjusted to reflect the actual uncertainties of state estimation in the long term.

The initial state error covariance matrix reflects the initial state-filtering accuracy, with little effect on the subsequent filter updating. The weight between Q and R determines the Kalman gain, which directly determines the impact that Q and R have on the state estimation. Therefore, the focus of the noise covariance reconstruction is partially shifted toward Q and R.

### 3.1. Adaptive Process Noise Covariance

Process noise covariance Q should be adjusted by the filter algorithm as it cannot be easily controlled directly unless adaptive factor α is used to tune the predicted state covariance. $P^{-}$ can be expressed as [26]:

(14) $$P^{-}=\alpha (\Phi \hat{P}\Phi^{T}+Qt)$$

where Φ denotes the state-transition matrix computed from the INS dynamic system, t is the discretization time, and $\hat{P}$ is the previous measurement updated state covariance. $\hat{•}$ and ${•}^{-}$ denotes the measurement updated parameter and predicted parameter, respectively. α is a scalar value, updated based on the weight between the covariance matrix of the predicted residual vector, denoted as ${\hat{C}}_{\xi }$, and the theoretical covariance matrix of the predicted residual vector, denoted as $C_{\xi }$. ${\hat{C}}_{\xi }$ and $C_{\xi }$ are expressed as [26]:

(15) $$\begin{array}{l} C_{\xi }=HP^{-}H^{T}+R\\ {\hat{C}}_{\xi }=\xi^{T}\xi \end{array}$$

where ξ is the innovation sequence and expressed as [25]:

(16) $$\xi =Z-H\delta x^{-}$$

with predicted state sequence $\delta x^{-}$.

Based on the Kalman filter principle, $C_{\xi }$ reflects predicted measurement error and theoretically equals to ${\hat{C}}_{\xi }$ in an ideal case (accurate dynamic model and measurement model, and their noise statistics) [26]. Assume measurements are measured without outliers and their noise probability density functions are accurate.

Let $\gamma =\mathrm{tr}({\hat{C}}_{\xi })/\mathrm{tr}(C_{\xi })$, tr(•) denotes the trace of a matrix. The adaptive function to determine the adaptive factors is expressed as [25,26]:

(17) $$\alpha =\left\{ \begin{array}{l} 1.0,\;\gamma \leq c_{0}\\ \frac{1}{c_{0}}\gamma ,\;c_{0}\leq \gamma \end{array}\right.$$

where $c_{0}$ is the corresponding empirical constant, which is typically equal to 1.5–2.0 according to Reference [25].

Under the condition of accurate measurements or accurate noise statistics, the KF time updating tends to be unstable when α > 1.0. $P^{-}$ is perturbed due to the dynamic model errors and should be tuned larger using α to ensure that $P^{-}$ closely reflects the actual situation.

### 3.2. Adaptive Measurement Noise Covariance

Adaptive modification of the noise covariance is a tradeoff between the convergence rate and filter stability. The filter error propagation reflects the accuracy of the state estimations to some degree. In an ideal KF application, tuning the noise models to yield consistent estimation errors and uncertainties can also produce stable state estimates that track their true counterparts [46]. α makes $P^{-}$ larger, and thus causes the filter gain value to increase, which increases the contribution of themeasurement outliers. Measurement outlier δξ can be considered as the unknown or unidentified uncertainty contained in the innovation:

(18) $$\tilde{\xi }=\xi +\delta \xi$$

According to Equation (17), predicted state error propagation $P^{-}$ can be increased β times without δξ, or inaccurately increased by α times with δξ and

(19) $$\begin{array}{l} \beta =\frac{{\left(\xi +\delta \xi \right)}^{T}(\xi +\delta \xi )}{\mathrm{tr}(C_{\xi })}/\frac{\xi^{T}\xi }{\mathrm{tr}(C_{\xi })}\\ \;=1+(2\delta \xi^{T}\xi +\delta \xi^{T}\delta \xi )/\xi^{T}\xi \end{array}$$

In the measurement updating procedure, Kalman gain K is calculated by [47,48]:

(20) $$K=P^{-}H^{T}{\left(HP^{-}H^{T}+R\right)}^{-1}$$

The measurement updating of the state estimate $\delta \hat{x}$ is formulated as follows:

(21) $$\delta \hat{x}=\delta x^{-}+K\tilde{\xi }=\delta x^{-}+K(\xi +\delta \xi )$$

A large proportion of the perturbations in the measurement is fed back to the state estimates because the corresponding gain value can be close to 1.0, subject to the large error magnitude of $P^{-}$.

If extreme measurement outliers occur, δξ tends to be extremely large. Then,

(22) $$|\frac{\delta \xi^{T}\delta \xi }{\mathrm{tr}(HP^{-}H^{T}+R)}\to \infty$$

Compared to the uncorrected R, ξ contains large-magnitude errors. Therefore, α tends to be extremely large. For a more intuitive analysis of the disadvantage of large measurement errors in $\tilde{\xi }$, we assume that α can be considered to tend to infinity. Based on L’Hôpital’s rule [49], the corresponding Kalman gain can be derived as:

(23) $$\begin{array}{l} \underset{\delta v\to \varepsilon }{\lim}K=\underset{\alpha \to \infty }{\lim}K\\ \;=\underset{\alpha \to \infty }{\lim}\alpha P^{-}H^{T}{\left(H\alpha P^{-}H^{T}+R\right)}^{-1}\\ \;=[\partial (\alpha P^{-}H^{T})/\partial \alpha ]{\left[\partial (H\alpha P^{-}H^{T}+R)/\partial \alpha \right]}^{-1}\\ \;=P^{-}H^{T}{\left(HP^{-}H^{T}\right)}^{-1} \end{array}$$

According to Equation (13), every block of the $HP^{-}H^{T}$ matrix consists of the linear transformation of skew-symmetric matrices, the determination of which equals to 0. Therefore, filter crashes can be produced due to singular-matrix inversion in the portion of the Kalman gain. This case rarely exists in general because the determination of R doesn’t equals to 0 and numerical issues cannot be caused when processing matrix inversion in Equation (23). However, when the measurement outlier magnitude is much larger than the magnitude of R, the contribution of R in gain calculation can be degraded, even ignored, and positive feedback, filter divergence, even the numerical problem still exist. Small errors in $P^{-}$ are relatively harmless; however, Equation (13) demonstrates that large $P^{-}$-matrix errors distort the Kalman gain matrix. R is often tuned by assigning state uncertainties that are substantially larger to an extent that nearly equivalent to $\xi \xi^{T}$, and the modified measurement noise covariance that contains unknown uncertainties $\bar{R}$ can be expressed as:

(24) $$\bar{R}=(\varsigma +\rho ){\left(\varsigma +\rho \right)}^{T}$$

where sequence ς indicates the inaccurate statistical uncertainty, and is empirically smaller than the actual measurement uncertainties. The sequence ρ represents the unknown measurement uncertainties, which are assumed to approximately correspond with the actual outliers. Thus, the adaptive factor is:

(25) $$\alpha =\frac{\gamma }{c_{0}}=\frac{1}{c_{0}}\frac{\xi^{T}\xi +2\delta \xi^{T}\xi +\delta \xi^{T}\delta \xi }{\mathrm{tr}(HP^{-}H^{T})+\varsigma^{T}\varsigma +2\varsigma^{T}\rho +\rho^{T}\rho }$$

If the hypothesis of Equation (22) is established, L’Hôpital’s rule can be used to determine α, and it turns out that:

(26) $$\alpha =\frac{\gamma }{c_{0}}=\frac{1}{c_{0}}\frac{\partial (\xi^{T}\xi +2\delta \xi^{T}\xi +\delta \xi^{T}\delta \xi )}{\partial (\mathrm{tr}(HP^{-}H^{T})+\varsigma^{T}\varsigma +2\varsigma^{T}\rho +\rho^{T}\rho )}=\frac{1}{c_{0}}\frac{||\xi |{|}_{2}+|\delta \xi |{|}_{2}}{||\varsigma |{|}_{2}+||\rho |{|}_{2}}$$

where $||•|{|}_{2}$ indicates two norms of a vector.

Due to the introduction of ρ, the contribution of measurement outliers is almost reduced with respect to α and $P^{-}$ afterwards. $P^{-}$ reflects the relatively precise uncertainty of the predicted state estimates and cannot be extremely large. Therefore, L’Hôpital’s rule cannot be used in the implementation of Equation (23), numerical issues concerning the inversion of ${\left(\alpha HP^{-}H^{T}+\bar{R}\right)}^{-1}$ are resolved, and the impact of positive feedback is attenuated because of the consideration of unknown measurement uncertainties in the Kalman gain determination. However, ρ is hard to be separated out; the most popular robust method in IAE is to tune α further by using an adaptive robust function that is expressed as [26]:

(27) $$\alpha =\left\{ \begin{array}{c} \;1.0\;,\;\gamma \leq c_{0}\\ \frac{\gamma }{c_{0}}\times (\frac{c_{1}-c_{0}}{c_{1}-\gamma }),\;c_{0}\leq \gamma \leq c_{1}\\ \;\frac{c_{1}}{\gamma }\;,\;c_{1}\leq \gamma \end{array}\right.$$

where $c_{1}$ is the corresponding empirical constant, which is typically equal to 4.5–8.5 [50].

Unfortunately, some drawbacks still exist for the following reasons:

- When tuning a Kalman filter, unknown uncertainties cannot be easily separated from the measurement noise. Although filter stability is ensured by assigning substantially larger state uncertainties, subjective assumption is introduced.
- The performance of measurement error equations in INS calibration is weakened when the measurement is in the steady state and cannot continually provide high-accuracy positioning and velocity results. Due to dynamic model errors and self-drawbacks of INS mechanization, INS cannot provide accurate state prediction. Due to low-accuracy a priori solutions or GPS outages, γ may be also extremely large even without a measurement outlier because Equation (27) cannot figure out the source of larger innovation deviation. Therefore, α can be less than 1.0, and the impact of $P^{-}$ matrix error is increased. According to Equations (21) and (23), a small $P^{-}$ matrix error produces unresponsive state estimates, while $P^{-}$ of a too-large error magnitude produces unstable, oscillatory state estimates [51].

**Figure 2:**
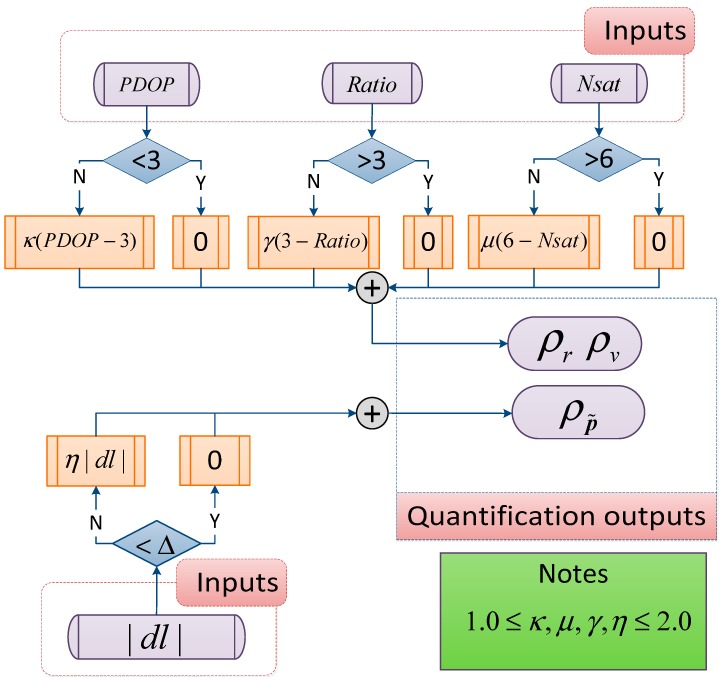
A quantification algorithm of Global Positioning System (GPS)-RTK reliability used in Equation (28) based on PDOP, number of valuable satellites, ratio, and length bias.

The contributions of $P^{-}$ and R determine the impact of dynamic and measurement models on the state estimation, respectively. Following Equations (17) and (27), the adaptive modification of $P^{-}$ relies on R, which demonstrates that focus should be placed on the measurement outlier detection and feedback. Hence, Figure 2 illustrates the proposed algorithm used to determine the ρ in Equation (24). In the two-antenna GPS/INS-integrated navigation application, the original R is modified based on filter measurement-updated covariance-solution processing by GPS-RTK, and reflects the filter accuracy of the GPS-RTK solution in detail ($\delta {\tilde{r}}_{\mathrm{GPS}}\in {ℜ}^{3\times 1}$, $\delta {\tilde{v}}_{\mathrm{GPS}}\in {ℜ}^{3\times 1}$, and $\delta {\tilde{p}}_{\mathrm{GPS}}\in {ℜ}^{3\times 1}$). $\rho \in {ℜ}^{3\times 1}$ is designed based on the position dilution of precision (PDOP) value, number of valuable satellites (Nsat), AR ratio and length bias (dl) of ${\tilde{p}}_{12}$ which can be obtained by GPS-RTK processing. These parameters can objectively reflect the quality of RTK solutions, which also improves for the modification of measurement noise covariance matrix and the determination of adaptive factors. To tune the measurement noise covariance matrix such that it closely aligns with the actual error magnitudes, the modified $\bar{R}$ is given by:

(28) $$\begin{array}{c} \bar{R}=E[ee^{T}]\\ e=\mathrm{diag}{\left[(\delta {\tilde{r}}_{\mathrm{GPS}}+\rho_{r})\;(\delta {\tilde{v}}_{\mathrm{GPS}}+\rho_{v})\;(\delta {\tilde{p}}_{\mathrm{GPS}}+\rho_{\tilde{p}})\right]}^{T} \end{array}$$

where $\rho_{r}=[\rho_{r}\;\rho_{r}\;\rho_{r}{]}^{T}$, $\rho_{v}=[\rho_{v}\;\rho_{v}\;\rho_{v}{]}^{T}$ and $\rho_{\tilde{p}}=[\rho_{\tilde{p}}\;\rho_{\tilde{p}}\;\rho_{\tilde{p}}{]}^{T}$.

The bounded dl(<Δ) is given by:

(29) $$dl=||{\tilde{p}}_{12}|{|}_{2}-||l_{12}|{|}_{2}$$

Figure 2 shows the algorithm to adaptively modify the measurement noise covariance matrix. The factors (κ,μ,γ,η) presented in the figure can quantify GPS-RTK reliability in detail to appropriately tune the measurement noise covariance matrix.

According to Figure 2, the adaptive measurement noise covariance matrix reconfiguration algorithm is formulated as the following equation. The algorithm is used to determine the driving state or measurement reliability based on the quantification results ($\rho_{r}$, $\rho_{v}$ and $\rho_{\tilde{p}}$), which can be equal or close to 0 when the GPS-RTK solution is reliable. Otherwise, state uncertainties larger than 1δ can occur in the instance when the measurement is less reliable. The factors mentioned in Figure 2, like PDOP and ratio, are directly related to positioning performance [52,53,54,55,56]; hence, the proposed measurement noise covariance matrix remodification method is more feasible and flexible compared with the adaptive robust method in Equation (27).

(30) $$\begin{array}{l} \rho_{r},\rho_{v}=\kappa (PDOP-3)+\gamma (3-Ratio)+\mu (6-Nsat)\\ \;\rho_{\tilde{p}}=\eta |dl| \end{array}$$

**Figure 3:**
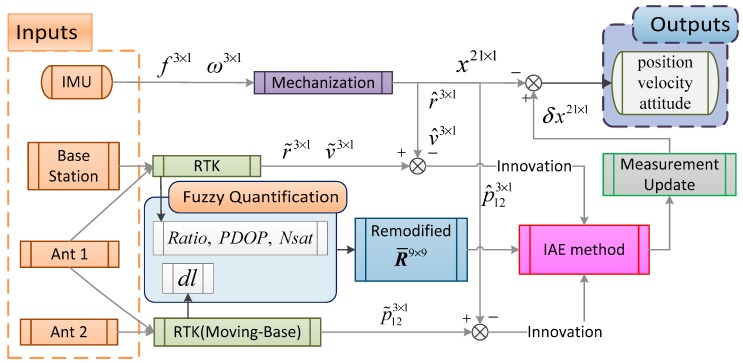
Overall flowchart of the proposed adaptive method in the proposed integration.

In summary, the overall flowchart of the two-antenna GPS/INS integration algorithm is shown in Figure 3. The IMU outputs are processed by the INS mechanization algorithm into navigation parameters, including position, velocity, attitude vectors, and the derived baseline vector $C_{b}^{n}l_{12}$ between the two antennas. Two GPS-RTK processing schemes were adopted to estimate the position and velocity vectors of Rover 1 and the two-antenna baseline vector $p_{12}^{n}$. In this research, the implementation involves a filter based on the proposed approach.

## 4. Field Test and Discussion

The proposed method was tested on POS with a NovAtel-OEM6 GNSS receiver (with two antennas) and the IMU with a gyroscope at the fiber level and the length between the antennas is 0.70 m. After INS calibration, the representative physical and operating specifications of the inertial sensors are summarized in Table 1.

To evaluate the performance of the proposed method in real environments, three data-processing schemes were designed in our data analysis:

- Scheme 1: two-antenna GPS/INS using a forward EKF.
- Scheme 2: two-antenna GPS/INS using a forward EKF with adaptive modification of the process noise covariance based on Equation (27).
- Scheme 3: two-antenna GPS/INS using a forward EKF with adaptive modification of both the process and measurement noise covariance, which is the proposed method.

Due to a Kalman filter being used in GPS-RTK processing, the same constant acceleration model [57] was adopted to properly evaluate the performance of the different schemes.

**Figure 4:**
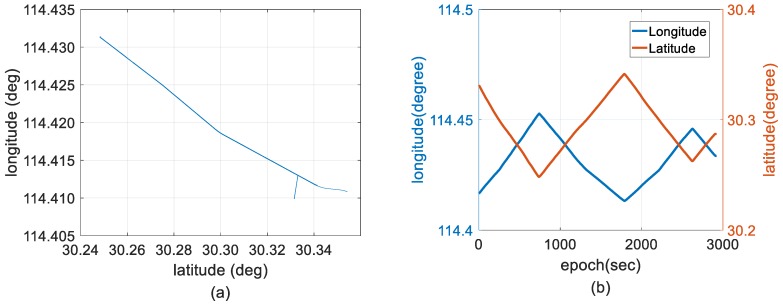
Reference trajectory. (**a**) Reference trajectory of dual-frequency ambiguity-fixed GPS/BDS/INS integration solutions processed by a backward filter with off-line Rauch–Tung–Striebel (RTS) optimal smoothing algorithm; and (**b**) reference longitude and latitude distributions for the whole duration.

In this research, only the single-frequency GPS dataset was processed using a forward filter to validate the performance of the proposed method for two-antenna GPS/INS integration, and GINS that data-processing software based on a Windows Forms operation, providing GNSS and GNSS/INS data post-processing functions, is utilized to provide reference solutions that using dual-frequency ambiguity-fixed GPS/BDS/INS integration solutions processed by a backward filter with off-line Rauch–Tung–Striebel (RTS) optimal-smoothing algorithm. RTS optimal smoothing algorithm is well-established in many applications, such as target tracking and state estimation; filter optimization can be accomplished by combining Kalman filter with RTS [58,59]. Figure 4 shows the reference trajectory with a distance of 37 km. Approximately 49 min of 1 Hz GPS/BDS data and 200 Hz INS data were collected.

### 4.1. Reliability of the Measurements

**Figure 5:**
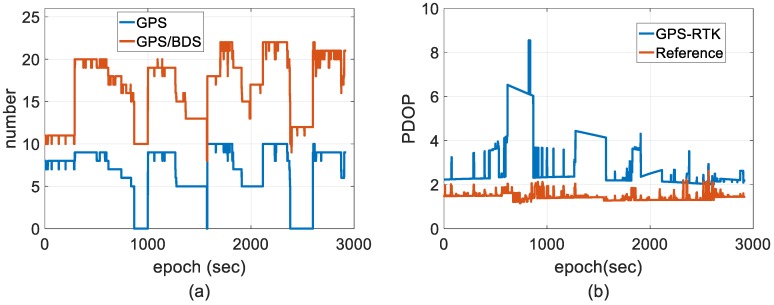
Distributions of valuable satellite number and PDOP. (**a**) Number of satellites and (**b**) PDOP.

To demonstrate the benefit of the reference solutions that can be qualified to evaluate the performance of the proposal implemented in harsh environments, the behavior of the PDOP, AR ratio and the number of valuable satellites are presented for the reference and tested solutions. Figure 5 shows the number of GPS and GPS/BDS constellation valuable satellites tracked by the rover receiver and PDOP behavior. The number of valuable satellites is nearly doubled by the introduction of BDS compared with using GPS only. The PDOP value for only using GPS was high; however, it was significantly reduced with the GPS/BDS integrated constellation after the deployment of BDS GEO and IGSO.

**Figure 6:**
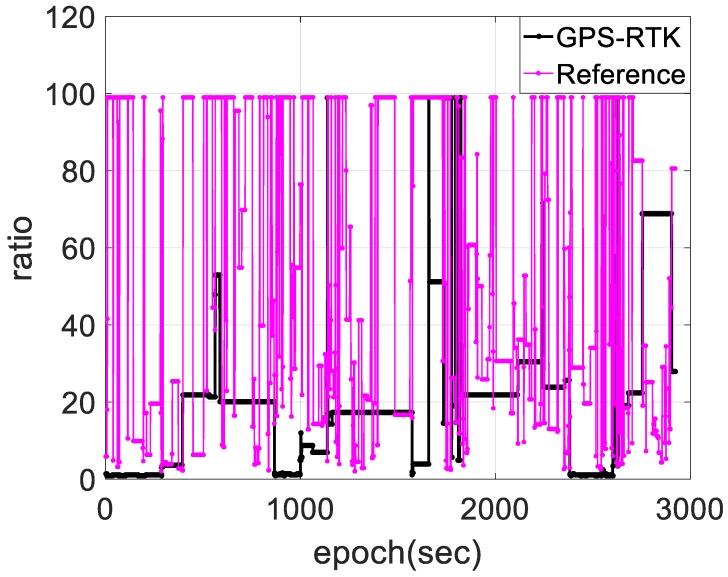
Ratio comparison between the referenced and GPS-RTK.

Figure 6 shows the AR ratio value distribution of the reference and those of GPS-RTK. The adoption of dual-frequency GPS/BDS/INS integration using a backward filter algorithm can improve the integer ambiguity resolution because better satellite availability from GPS/BDS and dual-frequency phase carrier adoption in data processing can further improve with a substantial decrease of ambiguity-float solutions, RTS smoother has an obvious accuracy advantage on filter, and INS provides strong constraints and short-term high accuracy to improve the accuracy of ambiguity-float solutions, position solutions with a centimeter error magnitude would be achieved. However, as a comparison, nearly 25% of the entire ratio solutions processed by single-frequency GPS-RTK using the forward-filter algorithm are fewer than three, and nearly 80% of the remaining solutions are considerably lower than the reference.

**Figure 7:**
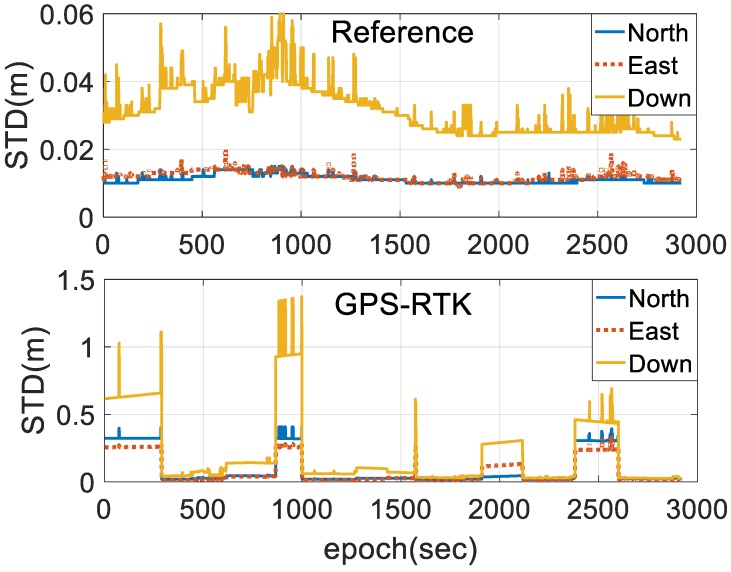
Position state estimate error of the referenced and GPS-RTK.

Figure 7 shows the positioning estimated state estimate error of the two schemes, illustrating that the stability of the state estimates is improved by the introduction of INS and BDS, and the state estimate error of the horizontal and vertical positions are bounded by 0.02 and 0.06 m, respectively, for the entire test durations. The solutions processed by single-frequency GPS-RTK using a forward filter are bounded by 0.05 and 0.2 m, respectively, when the system is in the steady state. However, the state estimate error of the horizontal and vertical position is increased by several decimeters under AR failure.

**Figure 8:**
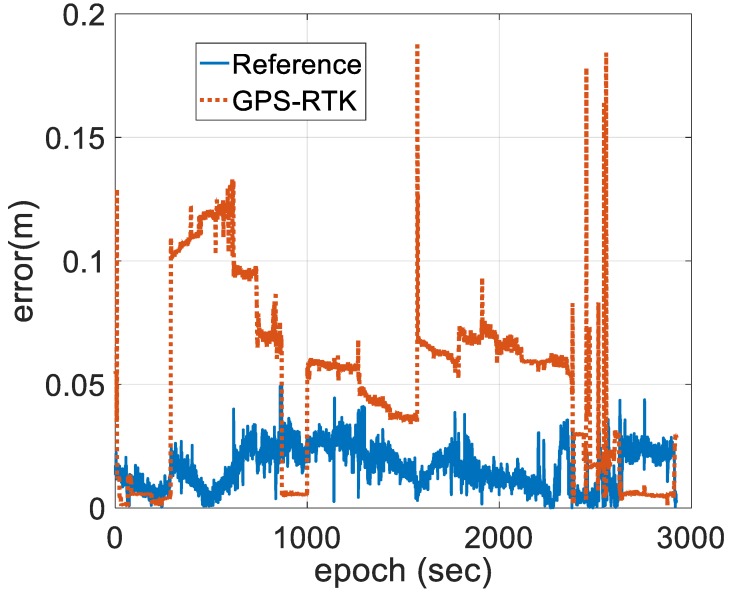
Two-antenna baseline length bias distribution.

Figure 8 compares the two-antenna baseline length errors between those processed by single-frequency GPS-RTK and the reference. The two-antenna baseline solutions processed by single-frequency GPS-RTK are often inaccurately estimated. The attitude state estimates in filter updating are not greatly influenced by occasional measurement outliers due to the filter robustness because of the detailed INS dynamic model and predicted uncertainty over a short period of time. However, its performance cannot resist the influence of long-term measurement outliers, since state prediction primarily relies on the mechanization algorithm of the INS, and a reliable dynamic model set cannot be easily established.

In our data processing, the initial parameters of the filter algorithm for the integration are determined based on experience. The initial position and velocity accuracy are determined based on the accuracy of the initial GPS information. The initial state estimate error of the gyroscope and accelerometer were set according to Table 1. Initial parameters, such as the process noise parameter, the initial state error covariance matrix, and the initial state vector, were identical for each scheme, obtaining better performance in comparison.

### 4.2. Experimental Results and Discussion

**Figure 9:**
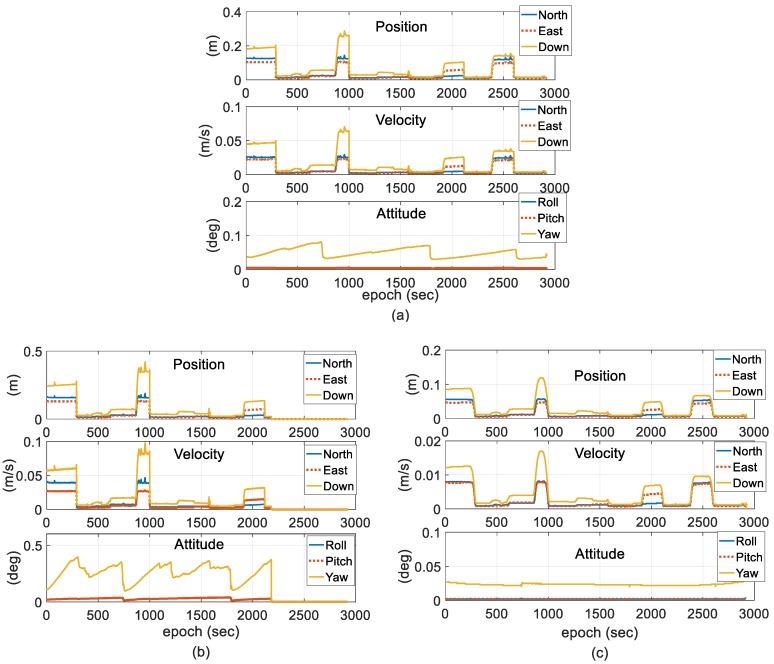
State estimate error for different schemes. (**a**) Scheme 1; (**b**) Scheme 2; and (**c**) Scheme 3.

The performance of the representative navigation parameters, such as position and attitude in terms of stability and accuracy, was compared between the reference solutions and tested solutions. Figure 9 shows the corresponding state estimate error for different schemes. Under the steady state for the GPS system according to Figure 9a, the 2-antenna GPS-INS using the EKF can accurately estimate the horizontal position (less than 0.02 m), the vertical position (less than 0.06 m), the horizontal velocity (less than 0.004 m/s), the vertical velocity (less than 0.06 m/s), the roll and pitch angles (less than 0.005°), and the yaw angles, which are periodically unobservable (less than 0.081°). However, when the GPS is in the unsteady state, the max state estimate error for the horizontal position and vertical positions are 0.15 and 0.29 m, respectively, the max state estimate error for the horizontal velocity and vertical velocity are 0.03 and 0.08 m/s, respectively, and no significant changes in the state estimate error of the attitude estimation are observed.

According to Figure 9b, which shows the state estimate error of Scheme 2, each state estimate error is increased in the long term compared with Scheme 1. Filter crashes occur from 2180 s to the end of the test. When using Scheme 2, the predicted process covariance is increased to reduce the impact of the dynamic model error on the state estimates, which increases the uncertainties of the state estimates according to the procedure of measurement updating in the filter. However, measurement with outliers can improve the innovation sequence and maximize the estimation deviation.

As shown in Figure 9c, Scheme 3 using the conventional EKF with adaptive modification of the process and measurement noise covariance has a better performance than the other schemes in terms of the attenuation of the state estimation uncertainty, which means the proposed adaptive method leads a better performance in filter accuracy and stability from the perspective of Kalman filtering. As the main advantage of the proposed scheme, the state estimate error of the proposed method is significantly decreased, even in the unsteady state for GPS measurements. Compared with Scheme 1, position state estimate error is decreased by approximately 55%, velocity state estimate error is decreased by approximately 50%, roll and pitch state estimate errors are decreased by nearly 60%, and yaw angle state estimate error is decreased by 70%. According to the comparison of the three schemes, as the main state estimate, the estimation of yaw angle has no periodic divergence for the entire duration of data processing.

The state estimate error reflects the state uncertainties and is different from the actual deviation. To evaluate the performance in accuracy, comparisons of the error magnitude with respect to the reference solutions among the three schemes and some statistical characteristics are given.

**Figure 10:**
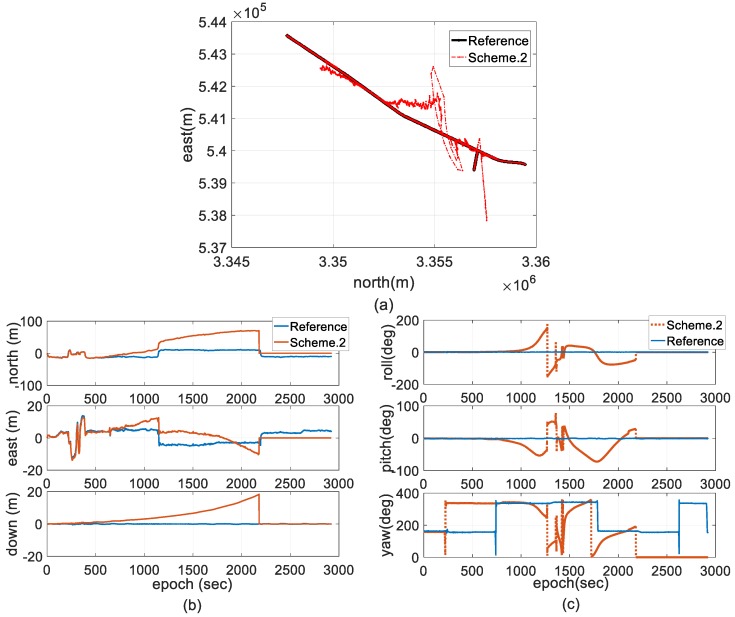
Comparisons of attitude and trajectory of Scheme 2 and the reference. (**a**) Attitude comparison; (**b**) velocity comparison; and (**c**) trajectory comparison.

Figure 10 compares the trajectory, velocity, and attitude solutions processed by Scheme 2 with the reference, which demonstrates that positive feedback exists in filter updating before filter crashes occur.

**Figure 11:**
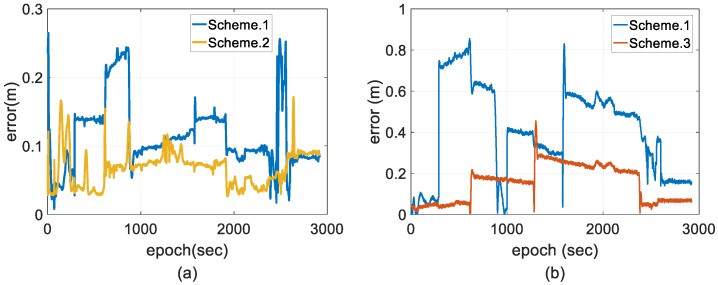
Comparisons of position error. (**a**) Horizontal; and (**b**) vertical.

**Figure 12:**
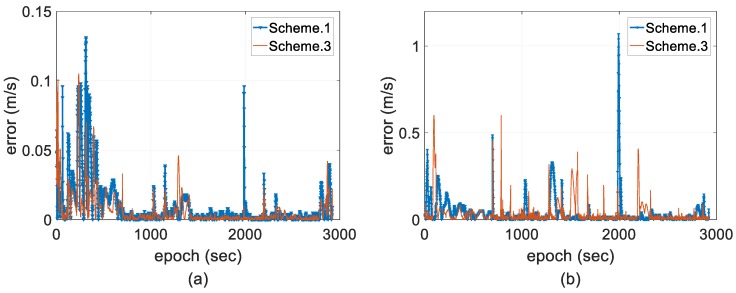
Comparisons of velocity error. (**a**) Horizontal; and (**b**) vertical.

**Figure 13:**
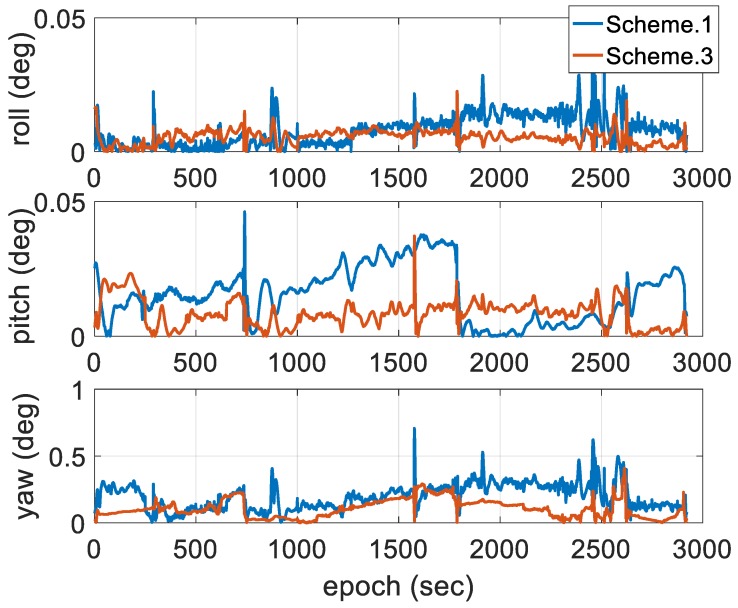
Comparisons of attitude error.

Figure 11 shows the error distribution of the horizontal and vertical positions, respectively. Figure 12 shows the error distribution of horizontal and vertical velocity, respectively. Figure 13 shows the error distributions of the roll, pitch and yaw angles, respectively. The following figures show that there’s no significant difference in the statistical results of velocity error; however, the position accuracy cannot remain in the stable state using the conventional EKF. The position solutions processed by Scheme 3 are of higher accuracy and stability than those of the schemes above.

**Figure 14:**
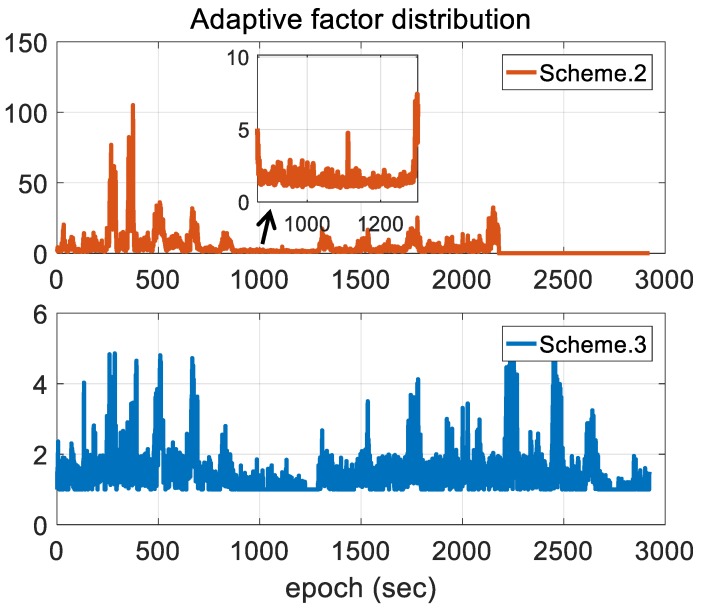
Comparison of adaptive factor distribution.

Figure 14 gives the adaptive factor results for the three schemes. It should be noted that we don’t discuss Scheme 1 since it uses conventional EKF without a corresponding adaptive reconfiguration on noise covariance. According to the figure, the impact of measurement outliers with respect to adaptive factor determination exists and the corresponding impact is more obvious when implementing EKF with IAE method. Conversely, Scheme 3 takes measurement unknown uncertainties into account by the measurement noise covariance reconfiguration in this research, significantly improving adaptive mechanization under measurement outlier conditions.

For better evaluation, the corresponding statistical characteristics in the whole period, such as the mean error (ME), the root mean square error (RMSE), and the max error (MAX), are gathered in Table 2 and Table 3. The probability of horizontal error less than 0.05, 0.10, and 0.15 m, and that of vertical position error less than 0.10, 0.20, and 0.30 m are gathered in Table 4 and Table 5. The results illustrate that the EKF fails to achieve a precise state estimation compared with the proposed method. The comparison of the RMSE illustrates that the proposed approach improves the stability considerably. The quantitative results demonstrate that the proposed adaptive modification for two-antenna GPS/INS integration is relatively reliable and robust.

**Figure 15:**
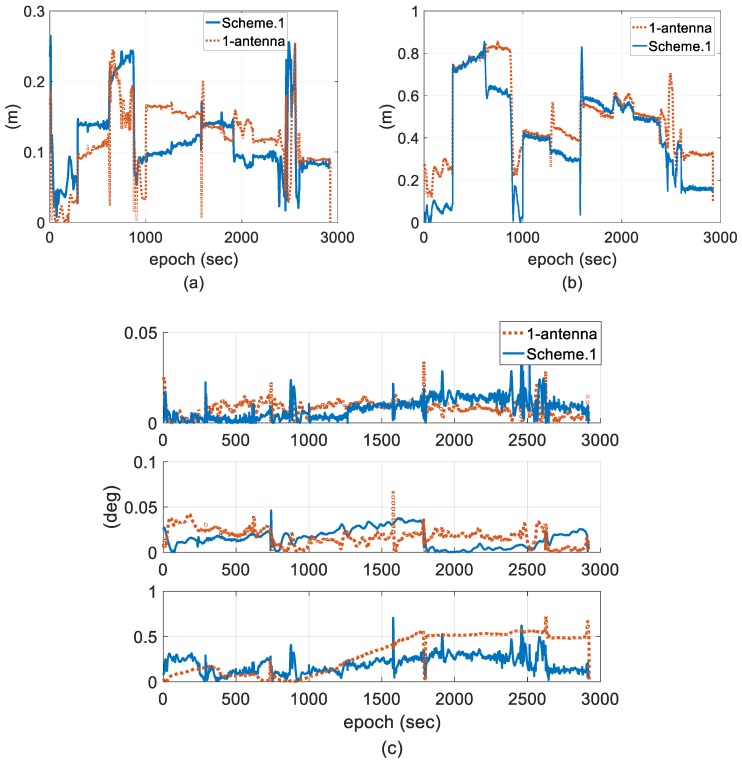
Error comparison of the one-antenna integration and the two-antenna integration using EKF. (**a**) Horizontal position; (**b**) vertical position; and (**c**) attitude.

For better analysis on the cost performance index (CPI) by using the two-antenna integration to improve stability and accuracy, Figure 15 gives the error comparison of the one-antenna integration and the two-antenna integration using EKF.

According to the theory of optimal state estimation, the accuracy of state estimates, especially attitude state estimates, can be improved because measurement redundancy is improved by using an augmented equation. We can see from Figure 15 that during a relatively steady measurement state, such as a length bias within 10 cm between the two antennas or a fixed AR, the proposed two-antenna integration generally performs better in terms of position and yaw accuracy; however, a negative effect is obvious under measurement outliers. Furthermore, there is no significant impact on roll and pitch because of the small error magnitude. In summary, the two-antenna GPS (or GNSS)-aided INS approach takes advantage of providing complete vehicle state, which improves filter performance [60], and we chose low-accuracy single-frequency GPS positioning solutions as measurement to better demonstrate the inoperability of IAE method under situations that innovation contains both measurement and predicted unknown uncertainties; then, the statistics of both measurement and state noise have to be adapted, which indirectly proves that the CPI of the two-antenna approach is improved by the proposed adaptive method even when we use single-frequency receiver.

## 5. Conclusions

To obtain high-accuracy and stable positioning and orientation solutions with two-antenna GPS/INS integration in harsh environments, this paper introduces a method for controlling the contribution of the filter dynamic model, and measurements by adaptive modification of noise covariance. To resist positive feedback and numerical issues resulting from the large unknown uncertainty of the measurement, the modification of the measurement noise covariance is adjusted using a measurement noise covariance matrix reconfiguration algorithm based on quantifications of the driving conditions. The performance of the proposed method has been verified in terms of accuracy and stability with a field vehicular test, and the following conclusions can be drawn:

- The conventional EKF adopted in two-antenna GPS/INS integration cannot give a comparative performance in terms of the accuracy and stability of the state estimation because the corresponding noise covariance cannot be tuned by the filter algorithm itself in the case of dynamic model error and measurement outliers in harsh environments.
- Adaptive modification of the noise covariance process depends on the assumption that measurements are accurate and stable or measurement noise follows well-known statistical characteristics. Under unknown measurement uncertainties, the innovation sequence can be distorted by measurement outliers, and the contribution of measurement outliers is increased, which results in a more serious issue concerning the positive feedback on the state estimation and even the instability of the Kalman gain computation.
- The proposed method not only considers the dynamic model errors but also appropriately tunes the contribution of the measurements on the state estimates based on the quantitative reliability of the GPS-RTK solutions in detail. Filter crashes and positive feedback are completely resisted. By using the proposed approach, the two-antenna GPS/INS-integrated navigation system maintains a stable and smaller error magnitude over the long term, which reveals that tuning measurement noise covariance based on measurement outlier detection and unknown uncertainty compensation plays a very important role in stable state estimation.

## Figures and Tables

**Table 1:** IMU specifications.

Parameters	Accelerometer	Gyroscope
Range	±10 g	±300 °/s
Random Walk	0.1 m/s/$\sqrt{h}$	0.03 deg$\sqrt{h}$
Instability	25 m Gal	0.5 °/h

**Table 2:** Statistical analysis of position and attitude estimation error in Scheme 1.

	Mean Error (ME)	Root Mean Square Error (RMSE)	Max Error (MAX)
Roll (°)	0.009	0.006	0.042
Pitch (°)	0.016	0.010	0.046
Yaw (°)	0.197	0.098	0.707
Horizontal Position (m)	0.120	0.180	0.397
Vertical Position (m)	0.411	0.221	0.856
Horizontal Velocity (m/s)	0.010	0.018	0.131
Vertical Velocity(m/s)	0.039	0.083	1.068

**Table 3:** Statistical analysis of position and attitude estimation error in Scheme 3.

	ME	RMSE	MAX
Roll (°)	0.005	0.003	0.023
Pitch (°)	0.008	0.005	0.037
Yaw (°)	0.106	0.068	0.408
Horizontal Position (m)	0.060	0.080	0.171
Vertical Position (m)	0.151	0.090	0.456
Horizontal Velocity (m/s)	0.007	0.013	0.105
Vertical Velocity (m/s)	0.034	0.067	0.600

**Table 4:** Statistical analysis of the horizontal position error probability.

Position Error	≤0.05 m	≤0.10 m	≤0.15 m
Scheme 1 (%)	2.00	10.75	51.87
Scheme 3 (%)	28.04	92.87	99.04

**Table 5:** Statistical analysis of the vertical position error probability.

Position Error	≤0.10 m	≤0.20 m	≤0.30 m
Scheme 1 (%)	12.01	25.30	31.77
Scheme 3 (%)	39.88	63.16	99.18
